# Incidence and associated factors for incidental prostate cancer among patients who underwent surgery for benign prostatic hyperplasia: first report from Somalia

**DOI:** 10.1007/s00432-022-04319-0

**Published:** 2022-08-29

**Authors:** Abdikarim Hussein Mohamed, Ismail Mohamud Abdullahi, Feysal Farah Warsame, Hussein Ali Mohamud

**Affiliations:** Mogadishu Somalia Turkish Training and Research Hospital, Mogadishu, Somalia

**Keywords:** Incidental prostate cancer, Benign prostatic hyperplasia, Transurethral resection of the prostate, Open prostatectomy

## Abstract

**Background:**

The incidence rate of incidental prostate cancer (IPC) differs significantly among the reported studies in the relevant literature. There is a scarcity of studies regarding IPC reported from Sub-Saharan African Countries, including Somalia. The present is the first study that evaluates the incidence and associated factors for IPC among patients who had surgery for benign prostatic hyperplasia at a tertiary hospital in Somalia.

**Method:**

This retrospective study reviewed the data of 538 patients with benign prostate hyperplasia, 464 patients who underwent transurethral resection of the prostate (TURP), and 74 patients with open prostatectomy (OP) over 5 years. A binary logistic regression model was used to investigate the association between perioperative factors such as age, prostate volume, total prostate-specific antigen (TPSA) levels, type of surgery, specimen weight, and the finding of IPC.

**Results:**

IPC was detected in 17.6%, 18.3% of TURP, and 13.5% of OP patients (*p* = 0.002). The mean age of the patients was 71.82 ± 7.4; IPC patients had a significantly higher mean age than the BPH group (74 ± 10.9 vs. 71.3 ± 10.8, *p* < 0.001). Sixty-two percent of the patients were T1b, while 57.8% had ISUP grade groups 1 and 2. Patients with T1a had significantly higher International Society of Urological Pathology (ISUP) grades 1 and 2 than those with T1b (69.4% in T1a vs. 50.8% in T1b, *p* < 0.001). Increased age, higher TPSA levels, low prostate volume, and specimen weight were independently associated with the finding of incidental prostate carcinoma (OR 1.978, 95% CI 0.95–1.60, *P *< 0.04; OR 1.839, 95% CI 0.99–2.02, *P* < 0.001; OR 1.457, 95% CI 0.7102.99, *P* < 0.001, OR 0.989, 95% CI 1.07–2.94, *P* = 0.01). IPC was most commonly managed by active surveillance (54.7%), followed by androgen deprivation therapy in 28.4%. The overall survival rate for a 5-year follow-up in the entire cohort was 79%. The cancer-specific mortality was 8.4%.

**Conclusion:**

The study findings revealed a higher incidence and cancer-specific mortality rate of incidental prostate carcinoma. T1b stage, higher ISUP grade, older age, and higher preoperative TPSA were significantly associated with the overall mortality and cancer-specific mortality rate. More than half of the cases were managed by active surveillance, and it is a safe management strategy, particularly in low-income countries like Somalia.

## Introduction

Prostate carcinoma (PC) is the second most commonly diagnosed cancer in men, accounting for 15% of all cancers (Yilmaz et al. 2022). It is the third leading cause of cancer-related deaths worldwide among older males and a global health issue (Li et al. 2019; Hussein and Al-khafaji 2021).

The diagnosis of PC by histopathological examination of resected prostate tissue previously assumed to be benign prostatic hyperplasia (BPH) is considered clinical T1 or incidental prostate cancer (IPC) (Guo et al. 2022). The incidence rate of IPC differs significantly among the reported studies in the relevant literature. The IPC rate among patients undergoing surgical therapy for benign prostatic hyperplasia without evidence of a prior PC diagnosis ranges between 3 and 17% (Abedi et al. 2020).

IPCs are clinically insignificant, a low-risk, and indolent disease in most cases. It can be subclassified as clinical stage T1a or T1b based on the percentage of cancerous tissue resected (Cuzick et al. 2021). According to several recent studies, cancers originating in the transitional zone have a more favorable prognosis than those originating in the peripheral zone. In contrast, others suggested unfavorable and aggressive clinical courses (Nergiz et al. 2021).

The management of IPC has been debated for decades. The best options are based on the patient's age, life expectancy, histopathological characteristics of the tumor, and PSA level following TURP (Ahmad et al. 2012). European Association of Urology Guidelines recommends watchful waiting (WW) or active surveillance for incidental pT1a and pT1b tumors if the Gleason score is six or less and the life expectancy of the patients is less than 10 years. However, radical prostatectomy is the recommended option for cases with T1b cancer and a life expectancy of more than 10 years, poorly differentiated tumors, or high PSA levels after TURP, according to EAU guidelines (Abedi et al. 2020).

To date, no previous studies reported from Somalia regarding the incidence and predictive factors for incidental prostate cancer in men who underwent prostate surgery. This study aimed to evaluate the prevalence and associated factors for incidental prostate cancer among patients who had surgery for benign prostatic hyperplasia at a tertiary care hospital and the only cancer center in Somalia.

## Method

This retrospective study reviewed the pathology reports of all patients who underwent surgery for benign prostatic hyperplasia over 5 years using the electronic medical records in the hospital information system (HIS). Patients with symptomatic prostate enlargement who underwent surgery for benign prostatic hyperplasia with a postoperative histopathology report were included in this study. Patients with known or existing prostate cancer or concomitant bladder cancer and those with incomplete data were excluded from the study.

Perioperative data, including age, prostate volume (cc) measured by transrectal ultrasound, total prostate-specific antigen levels (ng/ml), and type of surgery either transurethral resection of the prostate (TURP) or open prostatectomy (OP), were retrieved from the medical records. All BPH patients who are candidates for surgery were routinely screened for PC. A DRE suspicious for malignancy and/or a serum PSA of > 4 ng/ml were considered indications for transrectal ultrasound-guided prostate biopsy (TRUS).

Histopathological parameters, including specimen weight (g), finding of incidental prostate cancer, pT stage [subclassified as clinical stage T1a (< 5%) or T1b (> 5%) based on the percentage of cancerous tissue resected] according to 2017 TNM classification, and International Society of Urological Pathology (ISUP) grade group classification system of each IPC were evaluated. Gleason group scoring was used per the 2019 ISUP Consensus Guidelines on Grading of Prostatic Carcinoma. Perineuronal invasion (PNI) and lymphovascular invasion (LVI) were also recorded.

Due to financial reasons, patients cannot present for periodic follow-up appointments, and most lose their follow-up appointments. Still, we reached through a cellphone obtained from the hospital information system to gain information related to the overall 5-year survival rate and the cancer-specific mortality. In addition, predictors of overall mortality and cancer-specific mortality rate, including age, preoperative TPSA, T stage, and ISUP grade, were evaluated.

Ethical approval for this study was obtained from the institutional ethics committee of Mogadishu Somalia Turkish Training and Research Hospital (REF. MSTH-10616). All patients previously consented to use their medical and surgical data for research purposes.

Data analysis was performed using Statistical Package for the Social Sciences version 26 software (IBM Corporation, Armonk, NY, USA). Mean ± standard deviation was reported for continuous variables, while frequency and percentage for categorical variables. The Chi-square test and Student's t test were used to compare the variables. Bivariate analysis was used to determine the association between variables. *P* value < 0.05 was considered statistically significant. A binary logistic regression model was used to investigate the association between perioperative factors such as age, prostate volume, TPSA, type of surgery, specimen weight, and the finding of incidental prostate cancer.

## Results

This study included a total of 538 patients, 464 patients who underwent TURP, and 74 patients for open prostatectomy. All patients had complete preoperative investigations, including TPSA, prostate volume, and postoperative histopathology reports; thus, none were excluded. The time for patient inclusion in the study was between 30 June 2017 and 30 May 2022. Incidental prostate cancer was detected in 17.6% (*n* = 95 patients), 18.3% of TURP patients, and 13.5% of open prostatectomy cases (*p* = 0.002). The mean age of the patients was 71.8 ± 7.42; IPC patients had a significantly higher mean age than the BPH group (74.0 ± 10.9 vs. 71.3 ± 10.8, *p* < 0.001). The mean prostate volume and specimen weight was 74.0 ± 23.6 and 19.9 ± 3.31; IPC patients had significantly lower mean volume and specimen weight than the BPH group (70.9 ± 16.5 and 16.8 ± 2.24 vs. 81.9 ± 20.8 and 20.5 ± 3.62, *p* = 0.02, *p* < 0.001) (Table 1). The mean TPSA of the patients was 3.92 ± 3.61; IPC patients had significantly higher mean TPSA than the BPH group (4.64 ± 3.53 vs. 3.41 ± 2.82, *p* < 0.001). There was no significant association between age, TPSA, and type of procedure (*p* > 0.05) (Table 2).

**Table 1:** Baseline characteristics of BPH and IPC patients

Variable	Total	BPH	IPC	*P* value
Age	71.82 ± 7.42	71.3 ± 10.8	74.0 ± 10.9	< 0.001
Prostate volume	74.0 ± 23.6	81.9 ± 20.8	70.9 ± 16.5	0.02
TPSA	3.92 ± 3.61	3.41 ± 2.82	4.64 ± 3.53	< 0.001
Surgical method				
TURP	475	379	85	0.28
Open prostatectomy	76	70	10	
Specimen weight	19.9 ± 3.31	20.5 ± 3.62	16.8 ± 2.24	< 0.001

**Table 2:** Correlation between perioperative and histopathological findings and type of surgery

Variable	TURP (464)	OP (74)	*P* value
Age	71.9 ± 7.82	71.07 ± 7.23	0.22
Prostate volume	67.1 ± 11.4	90.0 ± 13.9	< 0.001
TPSA	3.62 ± 1.43	3.21 ± 1.82	0.32
Specimen weight	38.2 ± 12.7	57.4 ± 14.3	< 0.001
Incidental prostate cancer			
Yes	85 (18.3%)	10 (13.5%)	0.002
ISUP grade group			0.009
GG1	40	1	
GG1	13	1	
GG3	7	2	
GG4	10	4	
GG5	15	2	
pT stage			0.60
T1a	36	0	
T1b	49	10	
Perineuronal invasion			
Yes	29	3	0.04
Lymphovascular invasion			
Yes	18	1	0.06

Of 95 patients with IPC, 37.9% (*n* = 36) were pT1a, while 62.1% were T1b. All IPC cases detected during OP were T1b, while 57.6% of TURP were T1b. Regarding the ISUP grade group, most of the patients (43.0%) had GG1; 29.4% and 60.1% of TURP and OP patients had GG4 and 5, respectively (*p* < 0.009). Regarding the pT stage and ISUB grade group classification system of each IPC, patients with T1a had a significantly higher rate of ISUP grade 1 and 2 than those patients with T1b (69.4% in T1a vs. 50.8% in T1b, *p* < 0.001) (Table 3).

**Table 3:** Stage and ISUP grade

Variable	pT stage		*P* value
	T1a	T1b	
Grade group 1	18	23	< 0.001
Grade group 2	7	7	
Grade group 3	4	5	
Grade group 4	5	9	
Grade group 5	2	15	

Increased age, higher TPSA levels, low prostate volume, and specimen weight were independently associated with the finding of incidental prostate carcinoma (OR 1.978, 95% CI 0.95–1.60, *P* < 0.04; OR 1.839, 95% CI 0.99–2.02, *P* < 0.001; OR 1.457, 95% CI 0.7102.99, *P* < 0.001, OR 0.989, 95% CI 1.07–2.94, *P* = 0.01). At multivariate logistic regression model, type of surgery either TURP or OP was not associated with the finding of IPC (OR 0.768, 95% CI: 0.65–1.02, *P* = 0.25) (Table 4).

**Table 4:** Multivariate logistic regression model for perioperative factors and the finding of incidental prostate cancer

Variable	Odds ratio	95% CI	*P* value
Age	1.978	0.95–1.60	0.04
Prostate volume (cc)	1.457	0.71–2.99	< 0.001
TPSA (ng/ml)	1.839	0.99–2.02	< 0.001
Surgical method	0.768	0.65–1.02	0.25
Specimen weight (g)	0.989	1.07–2.94	0.01

Among 95 patients with IPC, most of the cases (*n* = 52, 54.7%) were managed with active surveillance, 28.4% (*n* = 27) were treated with androgen deprivation therapy (ADT), radical prostatectomy in 10.5% (*n* = 10), and radiotherapy for 6.34%. Patients treated with ADT (bicalutamide 50 mg and goserelin 10.8 mg) had T1b, and life expectancy < 10 years.

The overall survival rate for a 5-year follow-up in the entire cohort was 79.2%. The cancer-specific mortality in our study was 8.42%; seven out of eight patients with cancer-specific mortality were in the ADT group, had higher ISUP grade and T1b stage. The remaining patient was in the radiotherapy group. Two patients with radiotherapy and one patient with ADT returned with distant metastasis. Ten patients in the AS group and two in the ADT group died of other causes. T1b stage, higher ISUP grade, older age, and higher preoperative TPSA were significantly associated with the overall mortality and cancer-specific mortality rate (95% CI 0.926–3.791, *p* < 0.001).

## Discussion

There is a scarcity of studies regarding incidental prostate cancer reported from Sub-Saharan African Countries, including Somalia. Men of African ancestry have higher prostate cancer incidence, higher mortality rates, and worse survival than men of other ethnicities. They have been linked to several predisposing factors, such as socioeconomic, environmental, and genetic factors (Yuan et al. 2020). The incidence rates of IPC in African countries and other developing nations are underestimated because of the current diagnostic limitation. Most patients are diagnosed late because of a lack of awareness, screening, and early detection with proper documentation and a lack of urologic equipment and expertise. To the best of our knowledge, this is the first study to evaluate the prevalence and associated factors for incidental prostate cancer among patients who had surgery for benign prostatic hyperplasia reported from Somalia. In our study, incidental prostate cancer was detected in 17.6%, 18.3% of TURP patients, and 13.5% of open prostatectomy cases, with most patients being T1b. A retrospective study by Gunda D et al. included 152 patients who underwent prostatectomy for presumed benign prostatic enlargement. They reported a higher incidence rate of IPC of about 21.71% compared to our study and was independently associated with age 70–80 years and PSA levels > 10 ng/ml (Gunda et al. 2018). A 10-year retrospective study from Pakistan of 2,386 men aged 25–98 years with a mean age of 68.51 ± 9.22 years reported an incidence rate of about 10.7%, with 90.1% of the patients being T1b, which is remarkably higher than the stage we reported (Janjua et al. 2021). Among 295 men, 19% had a histopathological report indicating ICP (Yang et al. 2022). There is inconsistency in the literature with the reported incidence rates of ICP from surgical specimens from men with presumed benign prostate hyperplasia.

A systematic review and meta-analysis of 55 studies conducted by Cheng et al. aimed at reporting the incidence, associated factors, and the outcomes of IPC following endoscopic enucleation of the prostate concluded that older age, higher preoperative TPSA level, PSA density, smaller prostate volume and enucleated weight, and higher postoperative PSA velocity, were significantly associated with the finding of IPC (Cheng et al. 2022). Age remains the most common risk factor associated with prostate cancer development. The risk of prostate cancer increases with age and may occur as part of the aging process in men (Te et al. 2021). A retrospective study of 49,206 patients undergoing BPH surgery recruited from the Taiwan National Health Insurance Research Database compared resected specimen weight and subsequent incidental findings of prostate cancer. The authors reported that patients with a smaller resected specimen volume had a higher risk of prostate cancer with a hazard ratio (HR) of 1.221 (95% CI 1.035, 1.440; *P* = 0.0179) than those with a larger volume (Liu et al. 2019). Similar association between perioperative factors and the finding of incidental prostate cancer was observed at multivariate logistic regression model in the present study.

He G and colleagues conducted a comparative study of 1362 patients who received holmium laser enucleation of the prostate (HoLEP) and 1547 patients who received TURP for the diagnostic value of incidental prostate cancer between the two procedures for benign prostatic hyperplasia. HoLEP provided a significantly higher IPC detection rate than TURP (6.24% vs. 3.94%) (He et al. 2020). Capogrosso P and associates reported that HoLEP had a significantly higher chance of detection rate of incidental PC than TURP and Open prostatectomy (Capogrosso et al. 2018). Kizilkan and associates evaluated the IPC rate among 430 patients that underwent OP. The rate was 5.6%, which was much higher in elderly patients (Kizilkan et al. 2022). In our study, incidental prostate cancer was detected in 17.6% of the patients, and TURP had a significantly higher chance of detection rate of incidental PC than open prostatectomy cases.

The best optimal treatment options for incidental prostate cancer (IPC) are still debatable. Active surveillance, radical prostatectomy, radiation therapy, and androgen deprivation therapy are treatment modalities for these tumors based on the patient's age, life expectancy, histopathological characteristics, and PSA level following TURP. A German multicenter study (HAROW 2008–2013) of 210 IPC patients, of which 68 opted for active surveillance with a median follow-up of 7.7 years, reported 10-year overall survival of 83.8%, cancer-specific survival of 100%, and metastasis-free rate of about 98.4% (Herden et al. 2021). A 7-year study from the United States assessing the trends in diagnosis and management of IPC reported that patients diagnosed with T1a/b disease were significantly managed with active surveillance/watchful waiting and less likely to be treated with RP or radiation (Kord et al. 2022). In a cohort of 1020 patients, IPC incidence was about 5.6%; 73.6% was managed with active surveillance, 3.5% underwent radical prostatectomy, 10.5% had radiotherapy, and 12.2% had androgen blockade (İlktaç et al. 2021). More than 90% of IPC cases at diagnosis are International Society of Urological Pathology (ISUP) grade group 1, and active surveillance (AS) is now the standard of care and safe management strategy for most patients (Capitanio et al. 2022). A retrospective analysis of 18 and 42 patients with pT1a– pT1b PC, respectively, aimed at determining a 10-year survival rate, reported an 84% overall survival and 9.7% cancer-specific mortality for patients managed with AS, while 50% overall survival and 20% cancer-specific mortality in patients treated with ADT (Ahmad et al. 2012). In our study, IPC was most commonly managed by active surveillance, followed by androgen deprivation therapy. The overall survival rate for a 5-year follow-up in the entire cohort was 79%. The cancer-specific mortality in our study was 8.4%. Limited diagnostic capabilities such as multiparametric magnetic resonance imaging (mpMRI) and the inability to provide of curative management options, such as radical prostatectomy and radiotherapy, in addition to lack of awareness, screening, and early detection with proper documentation, are the leading principal causes of delayed presentation of the patients, which results in a decreased survival rate.

Although, the present is the first study that evaluates the incidence and associated factors for incidental prostate cancer among patients who had surgery for benign prostatic hyperplasia at a tertiary (the referral and the only cancer center) in Somalia. This study has certain limitations: first, it is a retrospective single-center study with small sample size. Second, no long-term oncological outcomes, including biochemical recurrence and metastasis, due to the patients' socioeconomic status and rural distribution, which limited the periodic follow-up appointments. Third, curative treatments such as radical prostatectomy and radiotherapy were not feasible in our country. Besides these limitations, we believe that the results of this study provide an essential contribution to global Incidental Prostate Cancer incidence and mortality.

## Conclusion

The present study revealed that the prevalence and cancer-specific mortality rate of incidental prostate carcinoma were high as in many low-income countries with limited urology resources. T1b stage, higher ISUP grade, older age, and higher preoperative TPSA were significantly associated with the overall mortality and cancer-specific mortality rate. More than half of the cases were managed by active surveillance, and it is a safe management strategy, particularly in low-income countries like Somalia.

## Data Availability

Data included in the manuscript.

## Abbreviations

ADT: Androgen deprivation therapy
AS: Active surveillance
BPH: Benign prostate hyperplasia
IPC: Incidental prostate cancer
HIS: Hospital information system
ISUP: International Society of Urological Pathology
mpMRI: Multiparametric magnetic resonance imaging
OP: Open prostatectomy
PC: Prostate cancer
PNI: Perineuronal invasion
LVI: Lymphovascular invasion
RP: Radical prostatectomy
SSA: Sub-Saharan Africa
TPSA: Total prostate-specific antigen
TRUS: Transrectal ultrasound-guided prostate biopsy
TURP: Transurethral resection of the prostate
